# The development of a novel sexual health promotion intervention for young people with mental ill-health: the PROSPEct project

**DOI:** 10.1186/s12913-024-10734-5

**Published:** 2024-03-01

**Authors:** Hayley Nolan, Brian O’Donoghue, Magenta Simmons, Isabel Zbukvic, Sophia Ratcliff, Alyssa Milton, Elizabeth Hughes, Andrew Thompson, Ellie Brown

**Affiliations:** 1 Orygen, 35 Poplar Road, 3052 Parkville, Melbourne, Australia; 2 Centre for Youth Mental Health, University of Melbourne, Parkville, Melbourne, Australia; 3 University College Dublin, Dublin, Ireland; 4 Faculty of Medicine and Health, University of Sydney, Camperdown, Australia; 5 Research Centre for Applied Health, School of Health and Life Sciences, Glasgow Caledonian University, Glasgow, Scotland; 6 Brain and Mind Centre, University of Sydney, Camperdown, Australia; 7 ARC Centre of Excellence for Children and Families over the Life Course, Sydney, Australia; 8 Division of Mental Health and Wellbeing, Warwick Medical School, University of Warwick, Warwick, United Kingdom; 9 Department of Psychiatry, Royal College of Surgeons, Ireland, Ireland

**Keywords:** Youth mental health, Sexual health, Comorbidity, Complex interventions, Participatory design

## Abstract

**Background:**

Young people with mental ill-health experience higher rates of high-risk sexual behaviour, have poorer sexual health outcomes, and lower satisfaction with their sexual wellbeing compared to their peers. Ensuring good sexual health in this cohort is a public health concern, but best practice intervention in the area remains under-researched. This study aimed to co-design a novel intervention to address the sexual health needs of young people with mental ill-health to test its effectiveness in a future trial undertaken in youth mental health services in Melbourne, Australia.

**Methods:**

We followed the 2022 Medical Research Council (MRC) guidelines for developing and evaluating complex interventions. This involved synthesising evidence from the ‘top down’ (published evidence) and ‘bottom up’ (stakeholder views). We combined systematic review findings with data elicited from qualitative interviews and focus groups with young people, carers, and clinicians and identified critical cultural issues to inform the development of our intervention.

**Results:**

Existing evidence in the field of sexual health in youth mental health was limited but suggested the need to address sexual wellbeing as a concept broader than an absence of negative health outcomes. The Information-Motivation-Belief (IMB) model was chosen as the theoretical Framework on which to base the intervention. Interviews/focus groups were conducted with 29 stakeholders (18 clinicians, three carers, and eight young people). Synthesis of the evidence gathered resulted in the co-design of a novel intervention consisting of an initial consultation and four 60-90-minute sessions delivered individually by a young ‘sex-positive’ clinician with additional training in sexual health. Barriers and supports to intervention success were also identified.

**Conclusions:**

Using the MRC Framework has guided the co-design of a potentially promising intervention that addresses the sexual health needs of young people with mental ill-health. The next step is to test the intervention in a one-arm feasibility trial.

**Supplementary Information:**

The online version contains supplementary material available at 10.1186/s12913-024-10734-5.

## Background

People who experience mental ill-health (such as psychosis, bipolar disorder, personality disorder, and affective disorders) have poorer physical health than the general population [1]. As a result, they die on average ten years earlier than the general population [2]. The last decade has witnessed a rapid increase in developing and testing interventions to address this disparity, with varying degrees of success. These interventions have predominately focused on increasing exercise, promoting smoking cessation, and improving diet [1]. What has remained missing from the physical health agenda is sexual health, despite having safe and satisfying sexual relationships being as much of a priority for people who experience mental ill-health than those who do not [3].

A disparity exists between this aspiration and reality, with research studies repeatedly reporting higher rates of high-risk sexual behaviours, unplanned pregnancies, sexually transmitted infections, sexual dysfunction, and violent relationships in individuals with mental ill-heath [4–6]. Increasing evidence has shown that younger cohorts (i.e., aged under 25) report similarly high rates of high-risk sexual behaviours as adult populations [7, 8]. The effects of engaging in high-risk sexual behaviours can be distressing and substantially impact an individual’s longer-term reproductive health and sexual relationships [9]. Sexual health promotion targeting this vulnerable cohort needs additional attention, particularly as young adulthood is when both the onset of mental disorders and engagement in high-risk sexual behaviour peaks [10, 11]. It is, therefore, a critical point to intervene and promote sexual health for this population.

The World Health Organisation (WHO) definition of sexual health [12] is broader than being free from sexually transmitted infections (STIs); rather, it defines sexual health as experiencing sexuality that is satisfying, positive, and respectful, as well as being free from exploitation and violence. This holistic view should guide any sexual and reproductive health intervention. Such targeted interventions, however, are lacking for young people experiencing mental ill-health [13] despite multiple factors making them a high-risk population. For example, this cohort is more likely to miss out on the sex education provided by schools due to lower attendance rates [14], has reported lower sexual self-efficacy [15], and are more likely to engage in sexting activities [16]. The development of a novel intervention that directly targets those most at-risk of experiencing poor sexual health (young people experiencing mental ill-health) within a service they are already accessing (youth mental health services) therefore has the potential to directly address these challenges.

This paper reports on what we believe is the first attempt to synthesise evidence with stakeholder engagement to produce a prototype intervention to address this need. The specific aims of this paper are to: (1) report the steps taken to co-design a novel sexual health intervention, (2) discuss the resulting themes garnered from the analysis of stakeholder interviews and focus groups, and (3) describe the proposed intervention. By addressing these aims, we present a potential solution that supports mental health services taking a step toward filling this critical healthcare promotion gap.

## Methods

### Intervention development framework

**Fig. 1:**
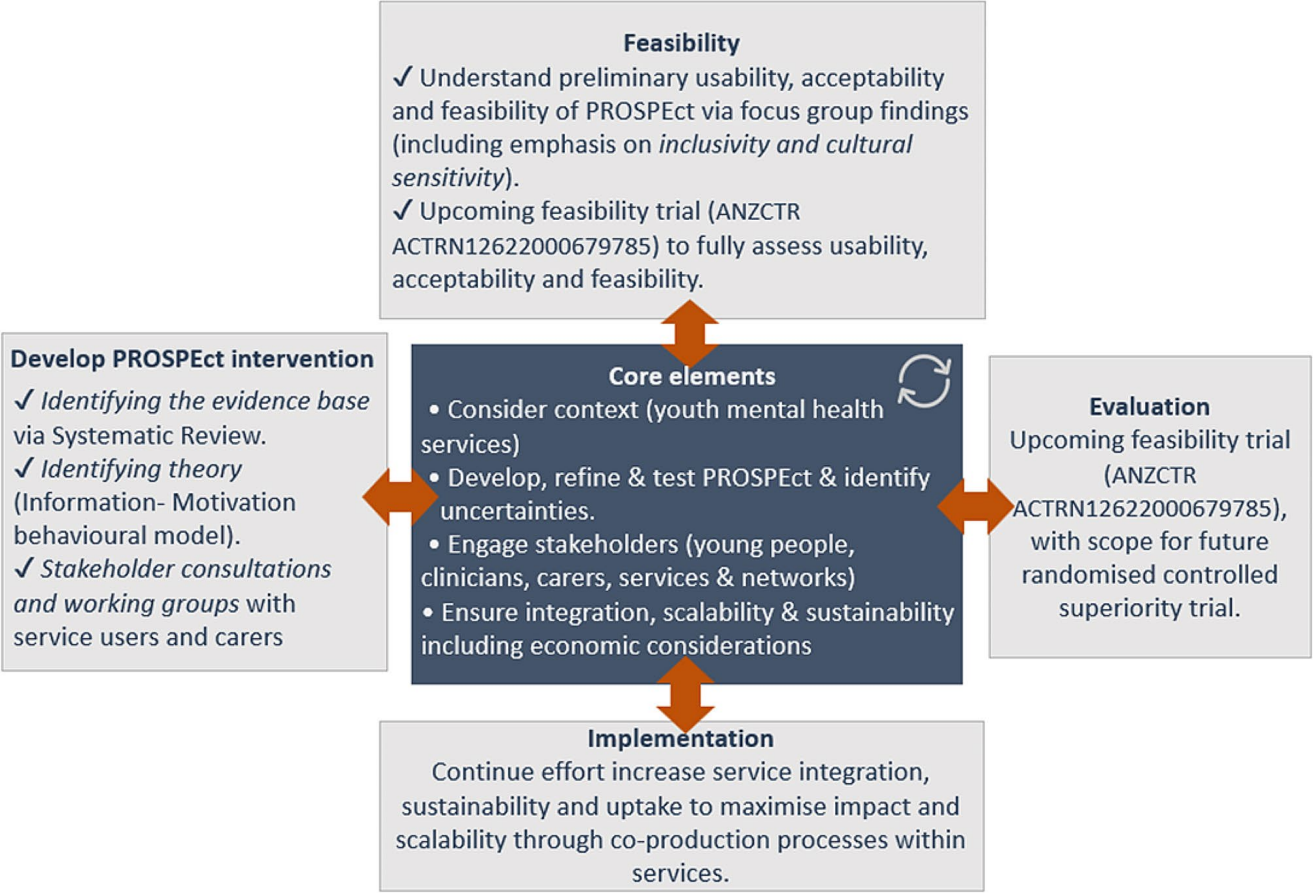
Mapping of current project onto the MRC framework for developing and testing complex interventions [17, 18]

The PROSPEct project followed the UK Medical Research Council’s Framework for developing and evaluating complex interventions [17, 18]. While originally devised to support research in the UK, this internationally applicable Framework is now increasingly being used by Australian researchers [19, 20], the country this current project was undertaken in. The Framework follows an iterative four-stage process to guide the development, feasibility, evaluation, and implementation of interventions to ensure they are acceptable, feasible, and effective within real-world healthcare settings. Notably, the process ensures that an intervention is based on the collation of the best available evidence, including observational research, stakeholder consultation, and expert opinion, as well as drawing on appropriate theory. Below we describe the methods undertaken to deliver each phase of the Framework through the PROSPEct research program to date, with this represented visually in Fig. 1.

### Development of the PROSPEct intervention

#### Identifying the evidence base

To ensure a comprehensive understanding of the available evidence-based sexual health interventions, we undertook a systematic review of behavioural and psychosocial interventions to promote sexual health in high-risk young populations. The aim was to identify the common content and delivery methods of previously tested interventions, and evaluate efficacy across high-risk cohorts. Outcomes of interest were indicators of holistic sexual wellbeing (e.g., condom use, attitudes to contraception, knowledge of risk). Participants were 25 years old or under and in one of the following high-risk and marginalised cohorts of young people; alcohol and other drug use; ethnic minority; homeless; justice-involved; LGBTQIA+ (lesbian, gay, bisexual, transgender, intersex, queer/questioning, asexual+); mental ill-health; or out-of-home care. The rational for taking this inclusive approach to cohorts targeted was the potentially applicable learnings from across the different at-risk populations.

In-depth findings from this review have been previously published [21], with the following conclusions influencing the current intervention development process. Twenty-six papers from 25 trials met the inclusion criteria, with all but one conducted in North America. Condom use was the most frequently reported outcome measure, along with knowledge and attitudes towards sexual health, with limited evidence for outcomes related to the quality of sexual relationships such as the presence of coercive control or sexual satisfaction. Also absent from previous research studies were measures of potentially risky contexts in which sexual activity occurs, for example, non-consensual sex, exploitation, controlling behaviours by intimate partners, and lack of social skills such as assertiveness and managing condom refusal in partners. Notably, changes in knowledge and attitudes did not consistently result in long-term behavioural changes, a finding in keeping with evidence from health behaviour change research, which shows that changes in attitudes and knowledge do not always correspond with changes in behaviour [22]. The majority of papers focused on the absence of high-risk sexual behaviours rather than taking a holistic approach that encompassed physical health, psychological wellbeing, and physical and psychological safety [21]. No one approach to addressing sexual health in at-risk youth stood out as a promising building block for the current intervention, rather, previous trials appeared dated and not rooted in contemporary views around sexual wellbeing.

Recommendations arising from the published review that influenced the development of the content and delivery methods of the current PROSPEct project included ensuring that future interventions focused on addressing sexual health more broadly than just the use of condoms and the absence of negative biological outcomes, for example, the presence of STIs or sexual dysfunction. Such an intervention should support behaviour change through ongoing support rather than a stand-alone information session. The review also consolidated which theoretical models had been used in the design of existing interventions, thus informing the theory used for the PROSPEct intervention and the next stage of the MRC Framework process.

#### Identifying theory

**Fig. 2:**
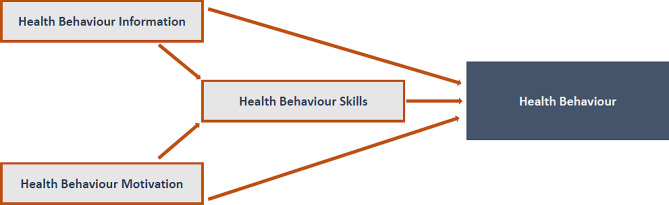
The components of the IMB model

The theoretical framework selected for the PROSPEct intervention was chosen because it had (a) guided the development of previous interventions in the field of sexual health in severe mental illness [3] and (b) was most frequently reported across studies identified in our systematic review [21]. While it was not possible to map theoretical model to intervention effectiveness through the literature review due to the lack of existing research, our previous work in the area (e.g. [23]) confirmed that the ‘Information-Motivation-Behaviour skills’ model of health behaviour change (IMB model, [24, 25], see Fig. 2) showed promise as a theoretical underpinning, particularly within severe mental illness populations, where misinformation and low self-efficacy can contribute to poor sexual health choices [26].

The IMB model proposes that while information is important for health behaviour change, an individual’s motivation to change is also critical. When considering sexual health specifically, it is likely that reflecting on the personal relevance of one’s sexual wellbeing will increase motivation to change. Using motivational interviewing techniques is one evidence-based way of achieving this shift [27, 28]. The model suggests that an intervention in this area should therefore focus on the following:

- **Information** (education) about the issues to improve knowledge of sexual wellbeing (e.g., HIV, STIs, contraception, risk behaviours, and what respectful, safe relationships should look like);
- **Motivation**, such as weighing up the pros and cons of contraception, mediating risk perception, and issues around consent;
- **Behaviour skills**, such as how to use condoms (if appropriate), problem-solving, and goal-setting skills.

#### Stakeholder consultation and working groups to refine the intervention

##### Participatory design

In mental health services and research, there is increasing momentum towards ensuring interventions are produced with, not just for, stakeholders [20, 29]. This ‘participatory design’ or ‘co-production’ approach [30] emphasises the importance of involving all stakeholders (i.e., those targeted by the intervention, involved in its design, or its delivery) during the development stage to ensure the resulting intervention meets the needs of all end users. ‘Co-production’ refers to the overarching approach used from outset to dissemination whereby individuals with lived experience guide the co-planning, co-design, co-delivery, and co-evaluation components of an intervention [30]. In the current part of the project we utilised co-design processes specifically. The development of the PROSPEct intervention involved four stages which aimed to include stakeholders in each part of the design process; (i) Stakeholder consultation round one, (ii) working group, (iii) stakeholder consultation round two, and (iv) inclusivity and cultural safety considerations.

##### Stakeholder recruitment across participatory design processes

Stakeholders were recruited from across Orygen clinical services which is a mental healthcare provider across North-western Melbourne, Australia, for young people aged 12–25. Young people recruited were aged 15–25 years and seeking treatment for mental ill-health across one of the Orygen clinical services (specialist or primary care programs). Carers were the primary caregiver of a young person who meets these criteria. Clinicians were recruited from Orygen Specialist or Primary Care Programs and held a range of professional backgrounds (psychologists, nurses, occupational therapists, peer workers, and GPs) as well as community sexual health organisations.

Ethical approval for developing the intervention using co-design processes and stakeholder input was obtained from the Royal Melbourne Health HREC (2020.093) including formal consent processes. Across all parts of the project, these were in line with Orygen and the Royal Melbourne Hospital standard procedures for gaining informed consent from individuals under 18 who could be considered mature minors. This refers to when a participant aged 15 to 17 is estranged from or has no contact with their parent or legal guardian, then consent may be obtained from the young person alone, when the young person has been assessed by a study doctor or delegate to have the capacity to provide informed consent. In this project it was acknowledged that a 15-17-year-old may not want to discuss their potential involvement with their parent or guardian due to perceived stigma around sexual health. We therefore gained ethics approval to extend the mature-minor clause to these individuals to avoid creating a biased sample. Interviews were undertaken by a young person with lived experience of mental health challenges with young people being interviewed separately to carers and clinician to ensure comfort when discussing the topic. The interviewer was supported by other members of the research team who were experienced clinicians and leaders in youth participation.

#### Stakeholder consultation round one

Semi-structured focus groups and interviews were conducted with 29 key stakeholders, including eight young people with mental ill-health, three carers, and eighteen clinicians who provide mental health or sexual health support to young people with mental ill-health. These initial focus groups and interviews aimed to explore broadly participants’ views about addressing sexual health within the context of youth mental health services and to establish preferences about key aspects of a potential sexual health promotion intervention, including content, setting, format, and delivery.

#### Working group

After completing analysis of the stakeholder consultations, a working group was convened with four young people who were youth mental health service users and advocates for mental health. This group met with the research team (HN, EB) throughout the development and refining of the intervention. The first draft of the novel intervention was shown and discussed across two meetings, with feedback sought on issues such as inclusive language and the content itself. This allowed the intervention to be adapted iteratively to reflect what would be most appropriate for young people with experience of mental ill-health.

#### Stakeholder consultation round two

A second round of stakeholder consultations were convened to allow for the devised intervention and its delivery methods devised to be modelled back to the participants involved in the first consultation. These involved three semi-structured focus groups to facilitate detailed discussions between a total of nine key stakeholders, including two young people with mental ill-health, one carer, and six clinicians providing mental health or sexual health support to young people with mental ill-health, were involved. These consultations were undertaken to gain insight into stakeholders’ thoughts on the proposed intervention developed through the above processes. The finalised version of the intervention was presented to the participants, and detailed discussions of all content material and planned methods for intervention delivery were facilitated. Feedback was incorporated into the intervention before moving to the next stage of the PROSPEct research program; feasibility and acceptability testing.

#### Inclusivity and cultural safety consultations

As with any intervention being developed or delivered, adaptations are required to target the needs of the local population they are serving [31]. Through the stakeholder consultations and youth working groups, the need to consider how best to address intersectionality within the intervention became apparent. Intersectionality describes how multiple social aspects of identity, such as gender, race, and sexuality, intersect or interact with each other [32]. Intersectionality is about seeing a person as a whole and encourages thinking beyond discrete labels that make up someone’s identity. Within the current context, the most prominent identities considered were identifying as a member of the LGBTQIA + community and from ethnic minorities. Utilising existing networks of clinical experts in gender-affirming and refugee care allowed for an iterative process of intervention refinement through ongoing consultation throughout the development process. This led to amendments to diagrams and language within intervention resources to ensure inclusivity was as wide as possible. These were recognised as critical elements in the current intervention as gendered language can impede communication, and the real– or perceived– inability to disclose gender identity safely may discourage gender-diverse individuals from seeking health care [33].

### Common features of qualitative phases of the study

For each of the qualitative stages of this project (stakeholder consultations (round 1 and 2) and the working group), the interviews and focus groups were digitally recorded, and recordings were transcribed verbatim. Data from each stage was analysed separately using thematic analysis [34] and content analysis [35] by two authors (HN and EB).

Thematic analysis [34] was used to focus upon the stakeholders views and experiences of addressing sexual health in youth with mental ill-health, with both inductive and deductive approaches utilised. This approach allowed us to explore both the responses to the research questions relevant to each stage of the intervention development, as well as emergent themes and issues that characterised stakeholders’ overarching experiences of the topic.

Content analysis [35] was used to deductively analyse data gathered regarding the delivery model of the intervention. This included gaining an understanding of preference for the format of the intervention (frequency, number, and location of sessions), and the delivery methods (location and intervention deliverer).

### Data analysis and synthesis of the intervention

The synthesis of the intervention arising from the intervention development stage of the MRC Framework (Fig. 1) will be presented in the following results section. This includes the results of the analysis (thematic [34] and content [35]) of the qualitative data elicited from stakeholder interviews/focus groups that are most relevant to informing intervention design and delivery. Also presented is the influence of the iterative stakeholder consultations on the end intervention.

## Results

### Thematic analysis of interviews

After completing the first round of stakeholder consultations, the following four themes were identified to guide intervention development. The first two, ‘Why is this still an issue?’ and ‘Who is responsible?’ represented barriers to addressing sexual health in young people with mental ill-health, while two others represented elements that potentially supported this area of work; ‘Knowledge is power’ and ‘Personal and societal change’.

### Theme 1: why is this still an issue?

Throughout the interviews, it became evident that despite recognition from all stakeholders that young people with mental ill-health need support with their sexual health and wellbeing needs, the promotion and treatment of these co-presenting health issues remained siloed. Topics discussed included barriers to accessing care, shame around discussing sexual health, and a lack of consensus on what constitutes ‘good sexual health’.

*“To be honest, I think a lot of the personal evaluation of sexual health for people begins and ends at things like STIs. Like, ‘I don’t have chlamydia, so my sexual health is 100%, and I’m fine’. Putting aside the mental and emotional impacts of it” (YP)*.

From the perspective of clinicians, broaching the topic of sexual health with their clients was considered challenging. This was related to multiple factors, including a lack of training, a disparity between recognising people with mental ill-health still identifying as sexual beings, or a fear of causing harm by broaching the subject.

*“I would feel very unsure of how to broach that topic…that is a real blind spot for me. I would want to avoid it completely because I wouldn’t want to bring up something that would be triggering as well” (Clinician)*.

### Theme 2: who is responsible?

There was a mixed response when discussions arose around who is responsible for ensuring sexual wellbeing within this cohort– young people, their carers, clinicians, GPs or somebody else entirely. Young people expressed views that despite wanting to take ownership of their health, they were often unsure of how and where to go for further support, at times only seeking care for physical concerns rather than psychosocial education. Some participants believed there was an onus on parents to educate and promote sexual wellbeing, which could be impacted by factors such as challenging family dynamics within this cohort. This often led to a reliance on education through peers, which some found challenging given they did not always have factual information to share. Additionally, media outlets, including social media and porn websites, were identified as frequent education sources. The problematic nature of this due to the potential lack of consent and the violent nature of some videos was frequently discussed.

*“Everything that they (young people) learn is from Pornhub. Pornhub is not a good place to get your sexual education. It is, if anything, the worst place” (YP)*.

Similarly, some stakeholders noted that schools were often where learning should theoretically take place but could be affected by common issues such as bullying. Several clinicians expressed that all healthcare providers actually have a responsibility to promote positive sexual health and should be more holistic in their approach, regardless of their profession;

*“Trying not to compartmentalise, you know, we’re talking about mental health, we’re talking about drug and alcohol issues, we’re talking about sexual health, they all intersect. And that’s what we have to remember.” (Clinician)*.

### Theme 3: knowledge is power

In contrast to the mixed response on responsibility and accountability within sexual health, there was a consensus that knowledge is powerful in this area. Young people can find it challenging to seek help if they are unsure of what is normal and what are deviations from the norm. Throughout discussions across focus groups, this was traced back to a lack of adequate sexual education for young people. One young person highlighted how current education often misses the mark;

*“We don’t want to just know how babies are made, because we’re going to have sex, we want to know how to have it safely” (YP)*.

In addition to inadequate sexual education, there is often a lack of proactive help-seeking from young people.

*“It (currently) is more of a reactive thing than an active thing, so instead of reaching out for knowledge or fact-checking, it’s ‘oh, I’ve noticed some symptoms” (Carer)*.

### Theme 4: personal and societal development

There was also an acknowledgement from clinicians that young peoples’ changing developmental stage affected what might be the most appropriate point to engage in sexual health education and healthcare. Ideally, this should occur when sexual identity is beginning to be explored, but if a young person experiences mental ill-health, this can impact emotional and cognitive capacities and thus the topic requires revisiting throughout their adolescence.

*“I think it is a good time when they’re questioning and they’re thinking about all of these things if somehow that could continue on…” (Clinician)*.

In addition to the changing development stages in a young person’s life, stakeholders highlighted that societal change is also happening, with sexual activity now commonly occurring online and on social media.

*“I think we get hung up in thinking about sexual activities being in person but there’s so much sexual activity happening in screens” (Carer)*.

This depicts the need for more robust sex education, with current curriculums not necessarily acknowledging how people engage in sexual activity in the 21st century.

Finally, multiple stakeholders agreed that healthcare services that have made efforts to become more holistic plays a positive role in young people engaging with services. This has included more online appointments and practices becoming more youth-friendly and inclusive.

*“I recently went to a GP clinic for specifically queer people and it was really nice” (YP)*.

Inversely, many young people expressed dissatisfaction with negative experiences in healthcare settings that had not changed alongside shifting societal norms. There was a recognition that accessing care through these services can cause more harm– particularly services with a one-size-fits-all approach and unwillingness to individualise care in any capacity.

### Content analysis of interviews

In this section of interview analysis, the aim was to understand how participants felt the intervention should be delivered in a youth mental health setting. Responses to the following broad question “can you share what you feel is the most appropriate mode of intervention delivery?” Factors that were focused on were; the most appropriate medium to use, whether sessions should be individualised or in a group setting, number of sessions, most appropriate length for these sessions and who is best placed to deliver the content.

Responses on these topics generally varied, for example, weekly vs. fortnightly sessions, individual vs. group sessions, school vs. mental health clinic setting, and face-to-face vs. online signifying heterogeneity in the area and a lack of cohesive evidence base on which to base practice in this area. Mixed responses were discussed with the working group which occurred after the first round of stakeholder consultations, and where necessary, the second stage of consultations. In this project there was a need to consider pragmatic elements throughout the design process, including physical location, personnel and resource limitations. Table 1 outlines the feedback from stakeholders on the different elements of format and delivery and how this feedback influenced the development of the intervention. For example, regarding the number and duration of sessions, participants suggested that between one and eight sessions lasting between 30 and 90 min. Further consultation supported the decision to create a four-session intervention of a flexible 60–90 min length.

**Table 1:** Development of the delivery model of the intervention

Topic	Stakeholder input	Findings from systematic review [21]	Influence on intervention development
**Format**: Online vs. offline	Overall consensus was an option for a mix of mediums, with face-to-face delivery being prioritized.	Most trials undertaken face-to-face with online mode of delivery used in one more recent study.	Face-to-face prioritized so that in-depth work can be done & YP body language read. Online sessions could be held if client felt more comfortable. Short online videos used as a learning resource.
**Format**: individual vs. group sessions	Individualised approach recommended as group session could be intimidating for a sensitive topic. Potential for group work to play a vital role in opening important discussions and providing an opportunity for social connection.	Most trials have tested group-based interventions. Groups used particularly with populations that were together already (e.g. justice involved youth, home based care).	Individual sessions to explore the core concepts within the intervention to build confidence. An optional group session ran monthly to discuss sexual health with peers in a safe space.
**Format**: number & duration of sessions	Between 1 and 8 sessions suggested. Minimising commitment from YP viewed as best. Length of session suggestions varied30-90 min.	Intervention length and duration very varied, ranging from 45 min to 8 h, delivered over one to 24 sessions.	One initial consultation session followed by four individualised sessions lasting 60–90 min selected as most consistent feedback. One optional 90-minute group session monthly proposed to be trialed.
**Format**: frequency	Fortnightly sessions were chosen by stakeholders over weekly to allow time for content to be absorbed.	Intervention frequency very varied, delivered over one day to 7 months.	Sessions delivered fortnightly to allow for time to avoid YP feeling overwhelmed.
**Delivery**: location	Private space, familiarity and easily accessible for YP. Classrooms in schools also suggested.	All but two trials took place in locations that the young people were already engaged in (e.g., mental health clinic, group home, drop-in centres), rather than at schools.	Classroom sessions were not feasible for this intervention due to ethics. Private interview rooms in spaces that the YP may visit for mental health services were chosen. Zoom chosen as ‘online’ location for telehealth appointments due to platform familiarity.
**Delivery**: person to deliver	A multidisciplinary approach of sexual health clinicians, mental health clinicians and youth peer workers were consistently suggested. Lived-experience frequently mentioned as beneficial quality.	Person who delivered intervention varied greatly by at-risk population and research group. Resources available within the context of the research project typically contributed to decisions.	Practical constraints contributed to current decision to use a young ‘sex-positive’ clinician already involved in the project (HN) who undertook further sexual health training. Youth peer workers with lived experience would be engaged to co-host the monthly group sessions.

YP = young person

Alongside these varying views on features of intervention delivery and format. there were consistent views on the need for it to be respectful, inclusive, individualised care delivered in a safe and supported location via a flexible approach.

### Intervention content and presentation

The final design of the PROSPEct intervention consisted of four sessions delivered one-to-one by a young ‘sex-positive’ clinician who undertook additional training in sexual health promotion provided through Sexual Health Victoria, a local Not For Profit, Government funded organisation. The content covered during the four sessions is outlined in Table 2 and is complemented by a workbook designed for participants to fill in during sessions and their own time. This content was delivered through a mix of mediums that aligned with the chosen theoretical model (IMB model, Fig. 2). This included psychoeducation around biology, contraception choices, consent, and STI testing (*information*); hands-on activities to encourage active learning, prompt discussions, and practice behavioural skills e.g. condom application (*skills/behaviour*); and reflective journaling, found in the workbook, to increase a young person’s motivation to improve their own sexual wellbeing (*motivation*). Youth-friendly activities to prompt conversations encouraged engagement, such as card sorts, ‘swiping left/right’ activities, and story completion tasks.

**Table 2:** Intervention structure

Session	Type	Duration	Purpose/format
1	Initial one-on-one **check-in session**	20–30 min	To help the clinician learn about a young persons needs and what is most important to them to address
2–5	One-on-one fortnightly **education sessions** x 4	60–90 min each	To include activities which help young person learn about: - basic biology; - contraception; - self-esteem and identity; - finding out what you enjoy; - consent; - communication skills; - being safer in the 21st century; - how media can affect our sexual wellbeing; - how to stay healthy; and - where to find help.
6	An optional **group session**	60 min	An opportunity for young people to chat to other young people who have taken part in the intervention once the four one-on- one sessions have finished.

Through consultation around inclusivity and cultural safety, we ensured that the language throughout the intervention was inclusive and appropriate for young people of all sexualities and genders. This includes referring to anatomy directly rather than gendering body parts, including content on gender identity and gender (e.g., gender unicorn), destigmatising conversations about asexuality, and the difference in sexual attraction and desire.

## Discussion

This paper describes the first attempt to co-design a sexual health promotion intervention specifically targeting the needs and wishes of young people who experience mental ill-health. The complexity required to carry out and subsequently report the processes undertaken could potentially be compounding the lack of previous co-design in this area, alongside the reluctance to discuss sexuality and sexual healthcare in clinical contexts.

Through the intervention development stage of the PROSPEct project, we identified key themes that are potential barriers and supports to addressing sexual health promotion in young people with mental ill-health. Regarding barriers, stakeholders felt sexual health continues to be under-addressed due to a combination of issues including shame, lack of awareness, and training, with no clear agreement on who is responsible for sexual health among young people, carers, and clinicians. In contrast, there was underpinning support for the idea that knowledge is power in this area– young people said they needed to know how to stay healthy, and without a solid knowledge base on what constitutes good sexual health and where to seek help, young people might not access care until there is a glaring physical concern. Lack of knowledge was also a barrier for this cohort of clinicians as well, with a recognition that they needed to know how to address sexual health as a part of holistic mental health care, similar to surveys of mental health nurses [36]. Finally, we identified that young adulthood was an ideal time to be tackling this health inequality as this is a period of transition, developing identity including sexual identity, and becoming more sexually active. There has also been a noticeable societal shift in recent attitudes, with technology and social media playing a key part in sexual behaviour [16] and sexual health education [37, 38].

The content analysis of the focus groups and interviews informed decisions around intervention content and presentation. Suggestions made by participants were reflected upon in the working group and second stage consultations with the final decision being made to trial a four-session, fortnightly one-to-one intervention delivered by a young clinician who took a ‘sex-positive’ approach to dealing with this area of healthcare.

The MRC Framework reinforces how critical the development stage of an intervention is. If not adequately considered, interventions are likely weaker, more problematic to evaluate, and less likely to be applied in practice [17, 18]. Understanding obstacles to accessing an intervention as early as possible is critical to successful and sustained intervention participation. We believe that there are several strengths associated with the development work that we have outlined in this paper. Firstly, we conducted a systematic review of behavioural and psychosocial interventions (detailed in this published article [21]) and two national surveys to establish baseline knowledge and attitudes toward sexual health among young people and clinicians (publication in preparation and thus detailed findings not expanded upon in the current article). Combining these scientific data with high levels of consumer involvement throughout the project (from input into grant and ethics applications through to the intervention development [39]) has ensured the development of an evidence-based intervention created *with* young people, not just *for* young people. This was ensured by undertaking working groups and second round stakeholder consultations throughout the process, both of which confirmed that the intervention was acceptable to those consulted. Integration of this co-design methodology is integral to mental health service reform currently being undertaken across Australia [40], and our current work in this area builds on this progress.

### Limitations

While the development of the PROSPEct intervention by utilising the MRC Framework for the development and evaluation of complex interventions [18] and co-production methods [39] represents a rigorous methodological approach, several limitations exist. Our attempts to recruit a diverse group of young people by age, gender identity, cultural and linguistic background, and stage of service use were limited by undertaking these activities at the time of the COVID-19 pandemic lockdowns in Melbourne, Australia. For example, representation of cultural and linguistic diversity was limited and full consideration of all faiths and cultural beliefs was not possible, potentially limiting the suitability of the intervention for some young people. In addition, it was not possible to host as many in-person focus groups as planned, potentially limiting the richness of the data that can be elicited within a diverse group-based setting. Despite these limitations, the comprehensive nature of development work undertaken here has strengthened the intervention design and increased relevance and coherence for young people in an area that has typically not involved young people [41].

## Conclusion

Following the MRC Framework has enabled the co-design of an intervention that addresses the sexual health needs of young people with mental ill-health, ready for testing its potential feasibility, acceptability, and effectiveness. Specifically, our subsequent research phase is conducting a one-arm feasibility trial to test the intervention (ANZCTR ACTRN12622000679785). Overall, the area of sexual health in mental ill-health requires considerable academic and clinical focus, particularly in the area of youth mental health. Training mental health clinicians to deliver sexual health promotion and interventions and iteratively adapting interventions to ensure the provision of inclusive care across diverse intersectional populations are areas of future need.

### Electronic supplementary material

Below is the link to the electronic supplementary material.

Supplementary Material 1

## Data Availability

Data analysed during the current study are available from the corresponding author on reasonable request.

## Abbreviations

ANZCTR: Australian New Zealand Clinical Trials Registry
GP: General Practitioner
HREC: Human Research Ethics Committee
IMB: Information Motivation Belief
LGBTQIA+: Lesbian, Gay, Bisexual, Transgender, Queer/questioning, Intersex, Asexual
MRC: Medical Research Council
UK: United Kingdom
