# Supplementary material for: The development of a novel sexual health promotion intervention for young people with mental ill-health: the PROSPEct project
Source: BMC Health Serv Res. 2024 Mar 1;24:262. doi: 10.1186/s12913-024-10734-5 (PMC10905889; doi:10.1186/s12913-024-10734-5)
Supplement: Supplementary file 1 — Supplementary Material 1 [file 12913_2024_10734_MOESM1_ESM.docx]

**Supplementary file 1:** **Focus group/interviews topic guide**

Interview schedule A - First round focus groups

1. What is meant by sexual health for young people

2. How do they evaluate their own sexual health

3. What triggers help seeking,

4. What are key elements of a sexual health intervention,

5. What is the most appropriate mode of intervention delivery (platform, timing, location, and personnel).

|  | **Specific questions** | **Prompts if required** |
| --- | --- | --- |
| Intro Q | Can you please tell us what you think is meant by ‘sexual health’ for young people? | - More than just the absence of disease, possibility of safe & pleasurable sexual experiences? |
| Transition Q | Can you tell us how you believe young people evaluate their own sexual health? | - Relationships? |
| Transition Q | What do you think triggers a young person to seek help regarding their sexual health? | - STI symptoms - Unplanned pregnancy - Need for contraception |
| Focus Q | What do you believe to be the key elements of a sexual health intervention? | - Education - sexual health problems & risky behaviours - Motivation – to improve own sexual health - Behaviour/Skills – assertiveness, using condoms correctly |
| Focus Q | With that in mind, can you share what you feel is the most appropriate mode of intervention delivery? | - platform (online v F2F) - timing (how frequent) - location (online – app? Or F2F – MH clinic, sexual health clinic, home, public space?) - personnel (MH clinician, specific sexual health clinician, peer support worker, case manager or somebody else. Carers present? ) |
| Concluding Q | Is there anything else that anyone feels we should have touched on today? Please feel free to share these thoughts. |  |

Interview schedule B - Second round focus groups

We will present the intervention we have designed and discuss proposed mode of delivery

1. What do you think of intervention we are proposing?
2. Are there any elements we should do differently?
